# Evaluation of temporomandibular disorders among dental students of Saudi Arabia using Diagnostic Criteria for Temporomandibular Disorders (DC/TMD): a cross-sectional study

**DOI:** 10.1186/s12903-021-01578-0

**Published:** 2021-04-26

**Authors:** Kumar Chandan Srivastava, Deepti Shrivastava, Zafar Ali Khan, Anil Kumar Nagarajappa, Mohammed Assayed Mousa, May Othman Hamza, Khalid Al-Johani, Mohammad Khursheed Alam

**Affiliations:** 1 Oral Medicine and Radiology, Department of Oral and Maxillofacial Surgery and Diagnostic Sciences, College of Dentistry, Jouf University, Sakaka, 72345 Saudi Arabia; 2 Periodontics, Department of Preventive Dentistry, College of Dentistry, Jouf University, Sakaka, 72345 Saudi Arabia; 3 Oral Surgery, Department of Oral and Maxillofacial Surgery and Diagnostic Sciences, College of Dentistry, Jouf University, Sakaka, 72345 Saudi Arabia; 4 Prosthetic Dental Sciences, College of Dentistry, Jouf University, Sakaka, 72345 Saudi Arabia; 5 Department of Oral Diagnostic Sciences, Faculty of Dentistry, King Abdulaziz University, Jeddah, Saudi Arabia; 6 Orthodontics, Department of Preventive Dentistry, College of Dentistry, Jouf University, Sakaka, 72345 Saudi Arabia

**Keywords:** DC/TMD, Anxiety, Dental students, Diagnostic criteria, Parafunctional behaviours

## Abstract

**Background:**

Temporomandibular disorders (TMD) are a broad category of conditions arising from the various components of the temporomandibular joint complex. Bio-psychosocial model is the most accepted theory describing the etiopathogenesis of TMD. Dental students are vulnerable to psychological disorders, including anxiety, depression, and stress. Hence, the aim of the current study was to evaluate the prevalence and possible risk factors of TMD among dental students of various academic levels and explore the association of TMDs with demographic, academic, and psychosocial parameters.

**Methods:**

A total of 246 students of a Saudi Arabia dental school were chosen for the study. After getting consent, all students were examined according to the Diagnostic Criteria for Temporomandibular Disorders, including Axis I and II components.

**Results:**

The overall cross-sectional prevalence of TMD was found to be 36.99%. Pain arising from the jaw, temple, and the peri-auricular area were the most commonly reported symptoms and elicited signs during examination. Among the pain-related TMD, myalgia was the commonest diagnosed condition, whereas disc displacement with reduction was found prevalent in the intra-articular disorder category. Female (OR = 1.94; *P* = 0.004), married (OR = 1.74; *P* = 0.04), and students in clinical academic levels (OR = 1.65; *P* = 0.03) were found to have significantly increased risk of TMD. Among the psychosocial parameters, anxiety (OR = 1.55; *P* = 0.04) and parafunctional behaviours (OR = 2.10; *P* < 0.001) were shown to increase the risk of developing TMD. Students with any TMD reported to have significantly higher pain intensity levels (OR = 1.68; *P* = 0.01) and jaw functional limitations (OR = 1.45; *P* = 0.008).

**Conclusion:**

Dental students, especially in clinical levels were shown to pose a higher risk of developing TMD, hence strategies such as academic counselling and objective evaluation via rubrics should be planned to modify the administration of the curriculum, training methods and evaluation process.

## Background

The temporomandibular joint (TMJ) complex consists of bone, cartilage, muscles, ligaments, and associated neurovascular channels supplying to the structures [1]. The disorders arising from these structures are also complex in nature with varied presentations such as pain originating from bone and muscles; displacement of the articular disc, and degenerative conditions related to bony components [2]. Autoimmune conditions, trauma leading to fracture and dislocation of the joint also adds to the spectrum of conditions affecting the TMJ [3]. Involvement of adjacent structures, such as referred pain to the ear, head area, and cervical involvement, add to the complexity of TMD and further contributes to disability in a subset of TMD patients [4]. They also bring limitations to mandibular movements, thus causing distress in performing daily activities [5].

The aetiology of TMD is multifactorial and not yet clearly understood. The factors that have been studied for their involvement in TMD are arranged into three categories, namely “predisposing”, “initiating” and “perpetuating” [6]. Occlusion is one of the controversial factors studied for TMD. Currently, it is not considered as the primary cause and its contribution might be limited to predisposing or perpetuating the existing condition [6, 7]. Conditions such as macro or micro trauma and situations leading to abnormal loading of the masticatory system are considered as the initiating factors. Oral behaviours and behavioural factors, including clenching of teeth, emotional disturbances and social factors are considered as perpetuating factors for TMD [8]. Psychological and certain pathophysiological processes are found capable to create a conducive environment for the development of TMD and hence, they are regarded as predisposing factors [9, 10]. At present, the bio-psychosocial model is considered as the most accepted model that explains the complexity of the TMD [11, 12]. With the increasing understanding of TMD, it is now considered as an entity of central sensitization syndrome (CSS), characterized by chronic pain [13]. The concept of CSS revolves around the phenomena of central sensitization (CS), where neurons in the higher centres undergo morphological changes leading to alterations in their functionality. These hyper excitable groups of neurons primarily work on amplifying the pain response by various means and thus affecting the pain behaviour [14]. Initially, these changes are supposed to arise from the peripheral damage or dysfunction, but once the area of CS is established, it becomes autonomous, thus explains the spontaneous, exaggerated and chronic nature of TMD pain. Eventually in the process of chronification, other components such as an autonomic nervous system, endocrine, descending pathways and cognition adds to the complexity in the pathogenesis of TMD [14].

The complex nature of the TMD and its presentation also pose a problem in its evaluation, as there is diversity in presentation and subjectivity in recording the conditions. Hence, in 1992, the Research Diagnostic Criteria/Temporomandibular Disorder (RDC/TMD) was introduced [15]. Subsequently, researchers used it while studying the prevalence of TMD in various populations [9, 16–18]. In this classification, Axis-I was made to assess the biological component and Axis-II was designed to assess the psychosocial aspect. Initially, it was largely accepted by the international scientific community, but later concerns were raised regarding its sensitivity and specificity [18]. Therefore, extensive studies yielded the revised version of the RDC/TMD, the Diagnostic Criteria for Temporomandibular Disorders (DC/TMD). The revised criteria (DC/TMD) improved the Axis-I diagnostic algorithm in order to increase the sensitivity and specificity and added few tools for Axis-II evaluation. Currently, the Diagnostic Criteria for Temporomandibular Disorders (DC/TMD) is considered as the gold standard for evaluating TMD [3].

Presently, studies are being carried out in varied population using Diagnostic Criteria for Temporomandibular Disorders (DC/TMD) [19–21]. A common observation made in the past studies was the preponderance of TMD among females and in the younger age groups [9, 16, 22, 23]. Previous studies have also pointed out the higher prevalence of TMD in university students, especially medical and dental undergraduates [19, 20, 24, 25]. As we can appreciate the complexity of the dental curriculum, it’s demanding nature and challenging structure, the amount of stress, anxiety, and distress are bound to increase [26, 27]. Hence, it will be also important to evaluate the academic related parameters such as grade point average (GPA) and academic level influencing the TMD prevalence. Thus, the aim of the current study was to assess the prevalence of TMD among dental students in Saudi Arabia. Furthermore, it was intended to find a possible association between TMD and demographic, academic and psychosocial parameters in dental students.

## Methods

### Study identification

A cross-sectional study was planned in a prospective manner. The study was conducted as per guidelines of strengthening the reporting of observational studies in epidemiology (STROBE) [28]. The study was carried out during January to May 2018 after receiving the approval from the ethics committee.

### Illustration about the sample

The prospective study was conducted at a dental school in Saudi Arabia. In the current study, two academic parameters, namely, academic level and academic performance (GPA) were considered. As per five-year curriculum of the dental school, the students of the 1^st^ and 2^nd^ year are considered “preclinical”, as they remain engaged in dental skills laboratories. However, the students of the 3^rd^, 4^th^ and 5^th^ year learn and acquire skills in clinics and hence are considered in the “clinical” category. Based on the GPA, the student’s academic performance was categorized as low GPA (< 3) and high GPA (≥ 3). All students aged 18 years and above with a male: female ratio of 1.4:1 was included in the sample. Additionally, students who were proficient in the English language were included in the study. Subjects with current dental pain conditions or a recent trauma to the face / neck or underwent treatment for any such conditions were excluded from the study. Subjects who reported with a recognizable psychological or neurological disorder or currently undergoing pharmacological treatment for any such conditions were also excluded from the study.

### Calibration of the examiners

Based on the clinical experience in consulting and treating TMD and orofacial pain, four specialists including two consultants each from the speciality of oral medicine and prosthodontics were selected to be the examiners for the study. To ensure standardization, all examiners underwent a self-directed training session aided with authentic study material and videos (downloaded from the official webpage of the International Network for Orofacial pain & Related disorders Methodology-INFORM) before the commencement of the study. During the session, they were provided with copies of the Diagnostic Criteria for Temporomandibular Disorders (DC/TMD) protocol of Axis-I, Axis-II and the diagnostic decision tree. After they were acquainted with the theoretical aspect, they underwent clinical sessions diagnosing patients for TMD. The clinical training was extended until they gained sufficient expertise and came to an agreement about the protocol and diagnosis. The assessment of inter and intra examiner reliability was calculated by cronbac’s alpha until a high level of agreement value (˃ 0.80) was achieved.

### Data collection tools

The Diagnostic Criteria for Temporomandibular Disorders (DC/TMD) Axis-I and Axis-II protocol is considered to have high reliability index value, and so considered as the gold standard [12], hence considered in the current study. The Axis-I protocol includes a symptom questionnaire and an English version of the clinical examination. Based on the diagnostic criteria and reference to the decision tree, the diagnosis was made [3].

The standard Diagnostic Criteria for Temporomandibular Disorders (DC/TMD) symptom questionnaire comprises of 14 questions which helps to reveal the history, duration and weather any functional activity can modify the complaint by either aggravating or relieving it. The major symptoms which were enquired included pain (Q1–4), headache (Q5–7), jaw joint noise (Q8), and regarding the locking of the jaw while opening (Q9–12) and closing (Q13–14). With respect to the duration of complaint in recent history, 30 days was considered as the benchmark while questioning about the complaint and its modifying factors [3]. The responses for all questions except, the description of the pain were recorded in the dichotomous nature. The second part of the Axis-I involved clinical examination to assess the location of pain/headache, provocation of the familiar pain upon palpation, incisal relationship, midline pattern, mandibular movements, TMJ noise, joint locking, and muscle tenderness. All examination findings were performed for both sides of the jaw. As described in the diagnostic criteria, combinations of positive responses from the history and examination components of Diagnostic Criteria for Temporomandibular Disorders (DC/TMD) Axis-I, were designated a diagnosis for the particular subject [3].

The Axis-II of Diagnostic Criteria for Temporomandibular Disorders (DC/TMD) is designed to assess the psychosocial status. In the current study, we undertook 5 standardized questionnaires namely graded chronic pain scale (GCPS) including characteristic pain intensity and pain-related disability, jaw function limitation pain scale (JFLS), patient health questionnaire-9 (PHQ-9), generalized anxiety disorder (GAD) and oral behaviour checklist (OBC) [3]. These questionnaires are designed to assess different aspects of a patient’s psychosocial element.

### Study protocol and data collection

**Fig. 1:**
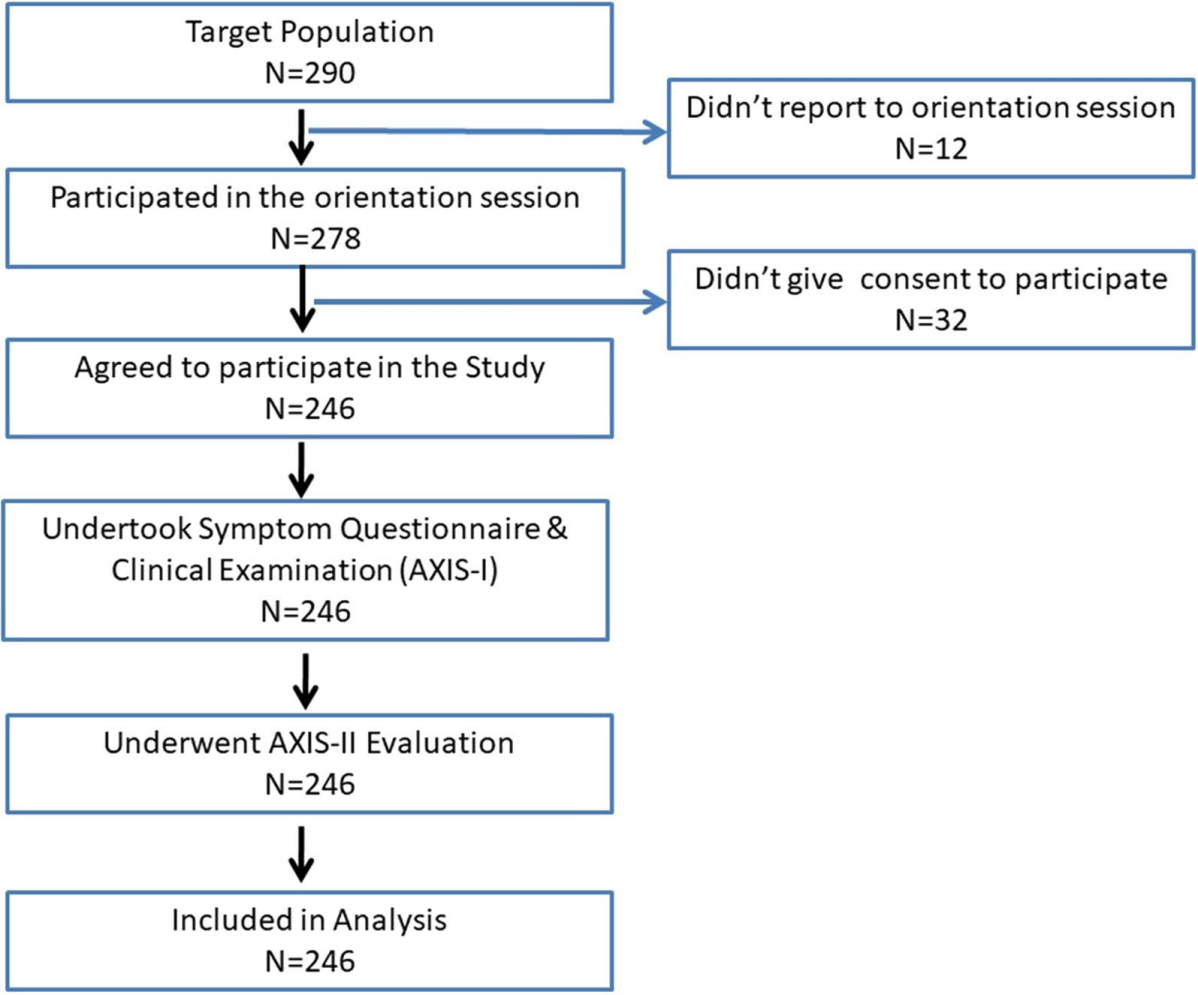
Flow chart of recruitment process of participants

All subjects were clearly explained about the objectives of the study and were not influenced in any manner to participate in the study. Participants were asked to give written consent before starting the data collection. Initially, an educational camp was conducted at the dental school where all undergraduate students were encouraged to attend. The camp was extended for 2 weeks where students from all academic years were contacted sequentially. They were informed about the symptoms and presentation of TMD. All students who attended the camp were motivated to come for the TMD screening session. Students who attended the screening session were evaluated according the Diagnostic Criteria for Temporomandibular Disorders (DC/TMD) criteria. A total of 246 dental students including 137 male and 109 female participated in the study and underwent Axis-I and Axis-II evaluation (Fig. 1).

The study involved 3-step protocol, where the first two steps involved the assessment of Axis-I and in the last step, evaluation of Axis-II was carried out. In the first step, the participants were given a symptom questionnaire. As per Axis-I protocol, students were subjected to the symptom questionnaire followed by clinical examination. Based on the responses from Axis-I and with the reference from the decision tree, the specific diagnosis of TMD was made. Lastly, all students were subjected to five structured questionnaire as a part of Axis-II evaluation.

### Data analysis

Considering the diagnosis, the sample was categorised into two groups. The group I (case group) consisted of 30 male and 61 female students with at least one TMD representing an overall prevalence of 36.99%. On the other hand, a total of 155 students with 107 male and 48 female did not report and TMD and hence considered as control (group II). The data was presented using numbers and percentages. For inferential analysis, the univariate and multivariate analysis was performed for the variables, where *P* < 0.05 was considered as statistically significant. All data analysis was performed with SPSS v.21. For the purpose of diagnosis of the psychosocial parameters, validated Diagnostic Criteria for Temporomandibular Disorders (DC/TMD) cut-offs were used [3]. As described previously, for computing univariate and multivariate analysis of the psychosocial parameter—GCPS, PHQ-9 and GAD-7, scores were dichotomized as “0” representing absent and “≥ 1” denotes present [14].

## Results

A total sample of 246 dental students across all academic levels from a dental school in Saudi Arabia was studied. The sample consisted majority of 20–25 years-aged subjects with male and female representing 55.69% and, 44.30% respectively. Additionally, more than half of the subjects (63%) belong to clinical oriented academic levels. Considering the GPA, about 82.92% of the sample represented ≥ 3 GPA, whereas only 17.07% of subjects has < 3 (Table1).

**Table 1:** Demographic characteristics of the study population

Variable	Response	*f* (%)
Age	< 20 years	64 (26.01)
	20–25 years	134 (54.47)
	> 25 years	48 (19.51)
Gender	Male	137 (55.69)
	Female	109 (44.30)
Marital status	Single	190 (77.23)
	Married	56 (22.76)
Academic year	Preclinical	91 (36.99)
	Clinical	155 (63)
Grade point average (GPA)	< 3	42 (17.07)
	≥ 3	204 (82.92)

Based on the assessment of subjects with the criteria of Diagnostic Criteria for Temporomandibular Disorders (DC/TMD) Axis-I, around 36.99% of subjects reported with at least one of the TMD. Pain arising from areas such as jaw, the temple, or in front of the ear was the most commonly (76.92%) reported symptom followed by joint noise (30.76%). On performing the clinical examination, pain/familiar pain was the most frequently recorded sign followed by alternations in the mandibular movement (64.83%) (Table 2).

**Table 2:** Frequency distribution of symptoms and signs reported by the subjects with TMD

Variable	Categories	Present *f* (%)
Overall prevalence of TMD (at least one TMD)		91 (36.99)
Symptoms*	Pain in Jaw, temple, in the ear or in front of ear	70 (76.92)
	Headache	17 (18.68)
	Joint noise	28 (30.76)
	Jaw locking	5 (5.49)
Signs**	Familiar pain	64 (70.32)
	Familiar headache	15 (16.48)
	Mandibular movement pattern	59 (64.83)
	Restricted mandibular movements	4 (4.39)
	Joint Noise	17 (18.68)
	Muscle tenderness	54 (59.34)
	Joint tenderness	13 (14.28)

*Data derived from the Diagnostic Criteria for Temporomandibular Disorders (DC/TMD) DC/TMD symptom Questionnaire

**Data derived from the Diagnostic Criteria for Temporomandibular Disorders (DC/TMD) DC/TMD clinical examination form

By combining the positive findings of the symptom questionnaire and clinical examination, the TMD diagnosis, namely, pain-related TMD, and intra-articular TMD were obtained. The distribution of individual conditions among the baseline variables are described in Table 3.

**Table 3:** Prevalence of TMD and frequency distribution of TMD diagnosis based on symptom questionnaire and clinical examination of Axis-I of Diagnostic Criteria for Temporomandibular Disorders (DC/TMD) among the variables

Variable	Present *f* (%)	Absent *f* (%)	Total (N)
Overall prevalence of TMD (at least one TMD)	91 (36.99)	155 (63)	246
Prevalence of pain-related TMD	68 (27.64)		
Prevalence of intra-articular disorders	25 (10.16)		

Diagnosis based on DC/TMD axis-I—combination of diagnostic questionnaire and clinical examination													
			Independent variables										
			Age			Gender		Marital status		Academic level		GPA	
		Total	< 20	20–25	> 25	Male	Female	Single	Married	Preclinical	Clinical	< 3	≥ 3
Pain-related TMD	Myalgia	62 (68.13)	12 (19.35)	31 (50)	19 (30.64)	21 (38.87)	41 (66.12)	24 (38.70)	38 (61.29)	22 (35.48)	40 (64.51)	37 (59.67)	25 (40.32)
	Myofascial pain with referral	6 (6.59)	0	4 (66.66)	2 (33.33)	1 (16.66)	5 (83.33)	4 (66.66)	2 (33.33)	0	6	4 (66.66)	2 (33.33)
	Arthralgia	12 (13.18)	2 (16.66)	6 (50)	4 (33.33)	3 (25)	9 (75)	5 (41.66)	7 (58.33)	4 (33.33)	8 (66.66)	5 (41.66)	7 (58.33)
	Headache attributed to TMD	14 (15.38)	3 (21.42)	6 (42.85)	5 (35.71)	3 (21.42)	11 (78.57)	6 (42.85)	8 (57.14)	5 (35.71)	9 (64.28)	6 (42.85)	8 (57.14)
Intra-articular disorders	DDWR	21 (23.07)	4 (19.04)	11 (52.38)	6 (28.57)	8 (38.09)	13 (61.90)	9 (42.85)	12 (57.14)	4 (19.04)	17 (80.95)	11 (52.38)	6 (28.57)
	DDWRIL	3 (3.29)	0	1 (33.33)	2 (66.66)	1 (33.33)	2 (66.66)	0	2 (66.66)	1 (33.33)	2 (66.66)	2 (66.66)	1 (33.33)
	DDWTRLO	1 (1.09)	0	0	1 (100)	0	1 (100)	0	1	0	1 (100)	0	1 (100)

*DDWR* disc displacement with reduction, *DDWRIL* disc displacement with reduction—with intermittent locking, *DDWTRLO* disc displacement without reduction—without Limited opening; individuals could qualify for more than one diagnosis

On multivariate analysis, female subjects were found to be at significantly (*P* = 0.004) higher risk (1.94 times) for developing TMD. Similarly, married subjects’ poses 1.74 times (*P* = 0.03) higher risk of having TMD, in comparison to their unmarried counterparts. Additionally, students at the clinical level were reported to have increased risk (*P* = 0.04) than their preclinical contemporises (Table 4).

**Table 4:** Univariate and multivariate analysis assessing the risk indicators for developing TMD

Parameter		Univariate analysis		Multivariate analysis	
		Odds ratio (CI)	*P* value	Adjusted odds ratio (CI)	*P* value
Age	< 20 years	Reference	–	–	–
	20–25 years	1.55 (0.65–1.96)	0.16	–	–
	> 25 years	1.86 (0.74–2.01)	0.29	–	–
Gender	Male	Reference	–	Reference	–
	Female	2.04 (0.85–2.56)	**< 0.001**	1.94 (0.42–2.34)	**0.004**
Marital Status	Single	Reference	–	Reference	–
	Married	1.95 (0.62–2.45)	**0.01**	1.74 (0.35–2.10)	**0.04**
Academic Level	Preclinical	Reference	–	Reference	–
	Clinical	1.84 (0.54–2.78)	**0.002**	1.65 (0.35–2.24)	**0.03**
GPA	< 3	1.22 (0.34–1.85)	0.08	–	–
	≥ 3	Reference	–	–	–

*CI* confidence interval

*P* values mentioned in bold are statistically significant

On evaluating the psychological components, subjects with TMD were found to have a significantly higher risk to possess parafunctional habits (*P* < 0.001) and anxiety (*P* < 0.04). Raised pain intensity level and altered jaw function are seen to be 1.68 times and 1.45 times higher in dental students with TMD as compared to students found with no TMD (Table 5).

**Table 5:** Univariate and multivariate analysis of Axis-II components of Diagnostic Criteria for Temporomandibular Disorders (DC/TMD) assessing the risk indicators for the development of any TMD

Parameter		Univariate analysis		Multivariate analysis	
		Odds ratio (CI)	*P* value	Adjusted odds ratio (CI)	*P* value
GCPS^a^	0	Reference	–	Reference	–
	≥ 1	1.94 (0.45–2.36)	** < 0.001**	1.68 (0.28–1.92)	**0.01**
JFLS-20^b^ item (any item ≥ 5 = 1)	0	Reference	–	Reference	–
	1	1.68 (0.30–2.15)	**0.006**	1.45 (0.10–1.85)	**0.008**
GAD^c^ (sum score 0–3)	0	Reference	–	Reference	–
	≥ 1	1.75 (0.32–1.96)	**0.02**	1.55 (0.22–1.84)	**0.04**
PHQ-9^d^ (sum score 0–4)	0	Reference	–	–	–
	≥ 1	1.64 (0.33–1.78)	0.56	–	–
OBC^e^ (sum score ˃16 = 1)	0	Reference	–	Reference	–
	1	2.42 (0.48–3.10)	** < 0.001**	2.10 (0.35–2.56)	** < 0.001**

*P* values mentioned in bold are statistically significant; *CI* confidence interval

Sum scores categorized (as per the Diagnostic Criteria for Temporomandibular Disorders) are as follows:

^a^Graded chronic pain scale (GCPS)—0-IV; grade I or II—low pain intensity without interference in the daily living; Grade III or IV—High pain and high interference, or moderate to severe disability

^b^Jaw functional limitation scale (JFLS)—any Item ≥ 5 = 1; remaining = 0

^c^Generalized anxiety disorder (GAD-7): 0 = no, 1 = mild, 2 = moderate and 3 = severe anxiety, respectively

^d^Patient health questionnaire-9 (PHQ-9): 0 = no, 1 = mild, 2 = moderate, 3 = moderately severe and 4 = severe depression, respectively

^e^Oral Behaviour checklist—sum score ˃16 = 1; remaining = 0

## Discussion

TMD is a complex condition with a multifactorial etiology. The major clinical presentation of these disorders is pain in the orofacial region of non-odontogenic origin [29]. The present study was carried out on dental undergraduate students of Saudi Arabia. Earlier studies done in Saudi Arabia had either used self-constructed questionnaires with university students [30] or employed only the TMD pain screener questionnaire in the general population [31]. Other researchers used RDC/TMD [18] for assessing the prevalence of TMD, or used fonseca anamnestic index (FAI) in combination with various other questionnaires for classifying the TMD [32–35]. The present study has an edge over other studies in Saudi Arabia, as it is first of its kind which has evaluated the male and female dental undergraduates for TMD using Diagnostic Criteria for Temporomandibular Disorders (DC/TMD). Since TMDs considerably affects daily functions, its early diagnosis, prevention, and therapeutic management are deemed necessary.

### Overall prevalence of TMD

Majority of previous studies have reported the prevalence of TMD among the general population ranging from 5–12% [3, 36], with few other studies reported even a higher prevalence of 25% [37] and 33% [38]. In the Saudi Arabian general population, the reported prevalence of TMD was 35% [31]. The heterogeneity in the population/race and the usage of different assessment tools by researchers could be the reasons for the variation in the prevalence of TMD.

The present study was conducted on dental undergraduates and reported the TMD prevalence of 36.99%. However, the higher prevalence of TMD has been reported in earlier studies (38% [39], 46.8% [33] and 62.8% [35]) conducted on similar population. This can be attributed to the inclusion of selective gender population and the use of FAI as the difference in the assessment tool [33, 35]. Along with this, few studies have used incomplete Diagnostic Criteria for Temporomandibular Disorders (DC/TMD) criteria wherein they have used online TMD screener questionnaire and have not conducted the clinical examination for making TMD diagnosis [39]. A probable reason for the observed prevalence of TMD among dental students in the current study was the complexity level of the curriculum and the demanding nature of the study patterns followed in Saudi Arabia. Additionally, dental students being more informed about the signs and symptoms associated with TMD, could have been more forthcoming in responding to the questions. Similar observation was reported by Zafar MS et al., wherein variation in the prevalence among the university students of different disciplines was found. The dental students had experienced more TMD compared to the pharmacy and medical students [40]. Contrary to this, Alamri A et al., reported a higher prevalence of TMD in pharmacy students compared to the dental undergraduates [32].

### Association of TMD with demographic parameters

A higher prevalence of TMD in females has been a consistently reported fact [20, 41]. Similarly, in the current study a significant female preponderance with 1.94 times higher risk for developing TMD was reported. However, Bagis B et al. has reported a 2.3 times higher risk of TMD in female [41]. Another study with adolescent sample had found the similar gender variation [42]. The higher prevalence of TMD in females can be attributed to various gender oriented variations including hormonal, anatomical and behavioural [43, 44]. Under the influence of estrogens, the laxity of the ligament increases during the preovulatory phase which further gets attenuated with the movement of TMJ leading to irritation of the TMJ joint [45]. Anatomically, it has been observed that usually males possess larger mandibular condyle compared to females which might have influence on the biomechanics of the TMJ [46]. Additionally, a higher bone mineral density in the condyle of female is correlated with osteoarthritis, which in turn could be one of the reasons for TMJ disorder [46]. Also, researchers have found that patients with TMD disorders mostly experience pain in other area which is considered as co-morbidity and it is more common in female [47, 48]. Lastly, coping strategies for stress have remained different among gender [45]. It has been observed that females experience a higher level of stress and depression as well they perceive higher pain compared to males [48].

### Association of TMD with academic parameters

Another interesting observation made in the current study was the higher prevalence of TMD among the students belonging to the clinical levels with 1.65 times more likely chances to develop TMD. This can be understood by acknowledging the fact that these students have acquired theoretical knowledge about the TMD as a part of their curriculum. Hence, they can relate well to the symptoms of TMD and so, thus responded to the questionnaire in a more responsible and informed manner. Inherent challenges, demanding patient care and apprehension about the career are some of the concerns which can be attributed to the students in the clinical levels [25–27].

Stress, somatic distress, and depression are seen as important etiological risk factors for pain related TMDs, thus these conditions have shown to be commonly associated with psychological distress [49]. In the present study, married students were reported with 1.74 times higher risk to develop TMD compared to their unmarried counterpart. In accordance with our results, Blanco-Hungría A et al., reported a higher prevalence of TMD among separated and divorced individuals followed by married, and least among the single individual [50]. Contrary to this, Han W et al., reported single females to have more TMD disorders compared to married contemporaries [51]. Although married individuals could have additional emotional support, prevailing social, economic and professional demands could challenge their threshold, and offset the balance of personal and professional life. Hence, the married individuals find difficult to find a balance between the personal and professional life [52].

### Association of TMD with psychosocial parameters

To assess the pain intensity and pain distress, graded chronic pain scale (GCPS) was used in the current study. It’s a tested and reliable tool used not only to measure the pain intensity, but also quantifies the deleterious impact of pain, while performing daily, recreational, social and work related activities in the last 30 days [3]. In the present study, it was observed that the students with a raised pain score on GCPS are 1.68 times more likely to have any form of TMDs. Pain reaction is a subjective and its threshold varies among individual [53]. It is also noteworthy to mention that the pain behaviour undergoes modulation due to central sensitization including altered responses in descending pathways and endorphins [13]. The phenotype of amplified pain is seen responsible for the persistence and perpetuating of TMDs. Although the pain attributed from the TMD usually starts as a somatic disorder, but it eventually progress to manifest as a chronic pain condition, where psychosocial components such as anxiety and depression plays an important role [14]. Likewise, in the current study the students with TMD were shown to have significantly higher pain intensity than students with no TMD. Our results are in agreement with the pervious study conducted with dental students of Saudi Arabia [35] and European continent [20].

Based on the conclusions drawn from the OPPERA study (orofacial pain prospective evaluation and risk assessment); a heuristic model was proposed. It describes the causal relationship and influence of various supposed risk factors including biological, environmental, genetic and psychological factors on the onset and progression of TMD [54]. According to this model, the two key phenotypes involved with the onset and graded progression of TMD are high states of “psychological distress” and “pain amplification”. The factors responsible for the manifestation of these phenotypes is seem to be up regulated due to the over-expression of their respective genes [55]. In order to evaluate this, Axis-II instruments for anxiety (generalized anxiety disorder-GAD), depression (patient health questionnaire-9-PHQ-9) and jaw functions (jaw function limitation pain scale—JFLS) were assessed. The students with a higher GAD score were shown to have 1.55 times higher risk for developing TMDs. Our observations restate the significance of the psychosocial component in the development of TMD. Student population generally seen to have higher anxiety levels compared to the adult population, as they remain in a constantly challenging environment [26]. Additionally, previous studies have shown dental students to have a higher level of stress and anxiety [56].

In the Diagnostic Criteria for Temporomandibular Disorders (DC/TMD), emphasis was laid on the patient’s assessment for not only the pain intensity and emotional component, but also disease-specific physical movement. With this intention, a new instrument—JFLS, was added to assess the impact of TMDs on the functionality of the jaws [3]. In the current study, dental students with TMDs reported to have higher functional limitations. The chronic TMDs no longer remain sensory, rather becomes a multisystem disorder with the involvement of the stomatoganathic system [14]. With the advancing chronification process, there is an alteration in the patient’s cognitive, emotional and behavioural aspect. In response, coping strategies such as psychological distress and self-isolation adopted by the patient are harmful and thus pose risk in the development of musculoskeletal disorder [55]. Likewise in the current study, students with high levels of JFLS were reported 1.45 times more likely to develop TMD.

The intention of including the oral behaviour checklist (OBC) was to evaluate the impact of destructive parafunctional behaviours. The checklist comprised of 21 questions pertaining to posture, motion and activities involving oral musculoskeletal structures including the jaws and tongue. The assessment differentiated between the behaviours performed during the sleep and awake state in the last month [3]. The students reported with masticatory muscle activity (MMA) were found to have 2.10 times higher risk to develop TMDs. Clenching/grinding teeth and chronic chewing gum were the most popular behaviours indicating a state of sub-conscious anxiety causing continual trauma to the masticatory system. [57]. This has shown to trigger sustained muscle contraction leading to fatigue and eventually causing chronic pain associated with TMDs [58].

### Limitations of the study

Despite the encouraging results, there are few limitations in the study as well. With a cross-sectional study design, the causal relationship between the variables cannot be established. Hence longitudinal studies with cohorts of all academic levels of different streams of education should be carried out. It will be also interesting to have multicenter study, so as to compare the pain behaviour. Although, the examiners of the current study were specialized in TMD, but their calibration process was self-instructed and based on available documents and videos. Nevertheless, a formal training/course for the potential examiners should be considered for raising the reliability of the diagnosis [59]. Although in the current study DC/TMD was adopted, however, the results of Patient Health Questionnaire-15 (PHQ-15) and pain persistency score (PSS) were excluded because of the incomplete/ inconsistence responses. This might be a limiting factor while comparing the current results with future studies.

## Conclusions

The present study concludes that TMDs are more prevalent in female dental students and students in clinical levels. Furthermore, pain was the most common sign and symptom associated with TMDs disorder in dental students. Modifications in curriculum, training and evaluation methods could help reduce the occurrence of TMDs in dental students.

## Data Availability

The datasets used and analysed during the current study are available from the corresponding author on reasonable request.
